# Liquid biopsy and blood-based minimal residual disease evaluation in multiple myeloma

**DOI:** 10.32604/or.2023.028668

**Published:** 2023-05-24

**Authors:** ALESSANDRO GOZZETTI, MONICA BOCCHIA

**Affiliations:** Division of Hematology, University of Siena, Azienda Ospedaliera Universitaria, Siena, 53100, Italy

**Keywords:** Myeloma, Liquid biopsy, Minimal residual disease

## Abstract

Novel drug availability has increased the depth of response and revolutionised the outcomes of multiple myeloma patients. Minimal residual disease evaluation is a surrogate for progression-free survival and overall survival and has become widely used not-only in clinical trials but also in daily patient management. Bone marrow aspiration is the gold standard for response evaluation, but due to the patchy nature of myeloma, false negatives are possible. Liquid biopsy and blood-based minimal residual disease evaluation consider circulating plasma cells, mass spectrometry or circulating tumour DNA. This approach is less invasive, can provide a more comprehensive picture of the disease and could become the future of response evaluation in multiple myeloma patients.

## Multiple Myeloma and Response Assessment

Multiple myeloma (MM)—the second most common hematologic malignancy after non-Hodgkin’s lymphoma—is characterised by monoclonal plasma cells that accumulate in the bone marrow and produce an abnormal monoclonal protein (monoclonal component [MC]) in the serum or urine that ultimately leads to organ damage [1,2]. MM can be preceded by precursor stages of monoclonal gammopathy of undetermined significance (MGUS) or smouldering MM that are asymptomatic. However, novel conditions of monoclonal gammopathies of clinical significance (MGCS) have been reported in which organ damage can be present, even with a small clone in the marrow [3–6]. Novel drugs, such as proteasome inhibitors and immunomodulatory and monoclonal antibodies, have elicited complete responses in MM patients [7]. Progression-free survival (PFS) and overall survival (OS) have nearly doubled in the last 20 years with respect to old chemotherapeutic regimens [8,9]. Progress has also been seen in peculiar forms of MM, such as plasma-cell leukaemia and extramedullary or IgM myeloma, although the prognosis remains dismal [10–13]. Due to the increased depth of response with the use of new drugs, the concept of minimal residual disease (MRD) evaluation has extended from clinical trials to clinical practice in many centres. MRD is measured by flow cytometry (next-generation flow [NGF]) or VDJ gene sequencing (next-generation sequencing [NGS]) on bone marrow aspirations.

Recently, the International Myeloma Working Group (IMWG) incorporated MRD assessment into the updated criteria for response in MM [14–17]. In particular, 10^−5^ was set as the ideal cut-off for MRD negativity with both techniques (NGF and NGS). Due to the patchy nature of bone marrow myeloma infiltration and the possibility of false negative results, imaging techniques derived from the evaluation of lymphomas, such as PET/CT or MRI, were included in the definition of response [18–21].

## Blood-Based Minimal Residual Disease Assessment

**Figure 1:**
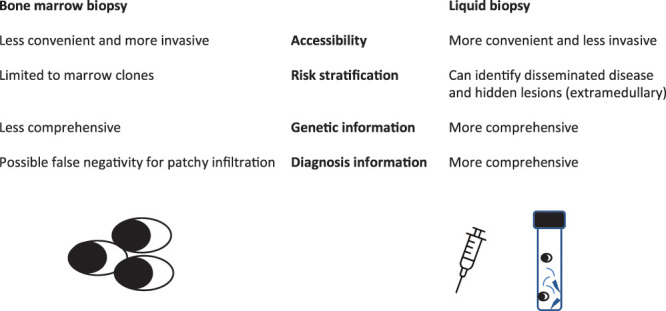
Different information given by bone marrow or liquid biopsy.

Advancements in MRD assessment have provided practical tools for patient response evaluation and have become the primary endpoint in many clinical trials. Nonetheless, the usual MRD evaluation is obtained from frequent marrow aspirations, which are invasive. Liquid biopsy (i.e., blood-based MRD analysis) could be important to increase MRD assessment because it could (a) allow for more convenient accessibility for routine MRD monitoring, (b) identify disseminated disease and hidden lesions and better risk stratify MM patients and (c) give complete genetic information on different clones that may be present and help find therapeutic strategies. While some techniques (mass spectrometry [MS] and circulating plasma cells [CPC]) are now very close to entering clinical routines, others utilising nucleic acid-based technologies are still experimental. MS methods can identify monoclonal proteins in peripheral blood (PB) and are an alternative to marrow-based tests for MRD (Fig. 1). These techniques are under development and could be important in the future.

## Mass Spectrometry

MS methods can identify the MC in PB and are an alternative to marrow-based tests for MRD. In particular, MS can (a) identify the MC with higher sensitivity than serum immunofixation, (b) differentiate monoclonal antibodies used for therapy from the MC and (c) detect the amyloidotic protein [21,22]. The MS mechanism of function relies on the unique mass of each immunoglobulin (based on the unique amino acid sequence). Several techniques are available, and each passes from the serum immunoglobulin enrichment, followed by reducing those to smaller constituents and calculating the final mass. The MC has a precise mass that is stable over myeloma history and can be measured sequentially for disease monitoring. Matrix-assisted laser desorption ionisation-time of flight MS (ALDI-TOF MS) is a technique that can rapidly detect the MC with more sensitivity [23]. Additionally, liquid chromatography MS is a technique that seems more sensitive than serum immunofixation in detecting the MC [24]. Other methods directly look for the MC from immunoglobulin heavy and immunoglobulin light chains, with a sensitivity 100 times more accurate than serum protein electrophoresis [25–27]. Studies are ongoing, and additional work is required, but preliminary results are encouraging, sometimes showing better sensitivity with MS than with marrow MRD analysis [28–31].

## Circulating Cell-Free DNA

Tracking tumour DNA mutations from patients’ blood has been done in different types of cancers [32,33]. There also seems to be a correlation between marrow and blood in terms of mutations and genomic alterations in the cell-free DNA (cf-DNA) of MM patients [34]. However, due to the shortage of cf-DNA in the blood, MRD assessment is still a challenge in MM. Deep sequencing (whole genome [WGS] and whole exome [WES]) seems to implement cf-DNA detection, but this technique is not currently adequate for MRD detection [35,36]. Studies comparing marrow and PB have shown a higher proportion of RAS/RAF mutations or clonal somatic mutations in PB [35]. Larger studies are needed to confirm the utility of such techniques.

## Circulating Plasma Cells

Circulating plasma cells (CPC**)** are determined by NGF, which identifies the antigenic characteristics of plasma cells, distinguishing normal from abnormal. In particular, EuroFlow described and standardised the methods of identification using two tube assays incorporating eight antibodies each: CD38, CD56, β2-Microglobulin, CD19, Anti-Kappa, Anti-Lambda, CD45 and CD138 and CD38, CD28, CD27, CD19, CD117, CD81, CD45 and CD138 (OneFlow™ *PCST* and PCD, BD Biosciences, San Diego, CA, USA) [14,17]. Recently, NGF has been used to investigate the frequency and number of CPCs in the blood of MM patients at diagnosis and has found their presence in all of them [37]. Additionally, smouldering MM and MGUS had a high percentage of CPCs in the blood (100% and 60%, respectively). CPC quantity has been reported as a surrogate for progression from MGUS to symptomatic MM (SMM). NGF was also applied to the study of MRD after therapy for the detection of CPCs in blood compared to marrow [37]. While NGF in the blood was able to identify CPCs in nearly 40% of patients that were serum immunofixation negative, marrow analysis was still confirmed as the gold standard because 40% of patients negative in the blood were positive in the marrow. Interestingly, those patients who became CPC negative in the blood had better outcomes than those who persisted in being MRD positive. Therefore, in the future, higher sensitivity could be important for improving prognosis. The number of CPCs in PB seems 100 times lower than in marrow. To increase the sensitivity of detection, large blood volumes are necessary for the assessment of MRD. A recent method with immunomagnetic beads targeting analysis seems to be crucial for the future. In fact, it seems to increase the sensitivity of MRD detection in blood by 10 times [38,39].

## Conclusion

The importance of liquid biopsy in MM is unquestionable and goes beyond its (understandable) preference by patients. PB is more convenient to gather than marrow, and myeloma MRD behaviour could be more well-defined if tested several times after therapy. The prognostic power of MRD could be reinforced, and MRD-driven therapy could be used. Although blood currently seems unlikely to reach the sensitivity level of marrow, it could soon be a surrogate for marrow evaluation (e.g., using blood until MRD becomes negative, thus decreasing the number of marrow aspirations). Further studies are needed to improve the sensitivity and assess the clinical utility of blood MRD.

## References

[1] Siegel, R. L., Miller, K. D., Fuchs, H. E., Jemal, A. (2021). Cancer statistics. CA: Cancer Journal Clinicians*,* 71 *(*1*),* 7–33. 10.3322/caac.21654; 33433946

[2] Gozzetti, A., Candi, V., Papini, G., Bocchia, M. (2014). Therapeutic advancements in multiple myeloma. Frontiers Oncology*,* 4*,* 241. 10.3389/fonc.2014.00241; 25237651 PMC4154387

[3] Kyle, R. A., Thernau, T. M., Rajkumar, S. V., Larson, D. R., Plevak, M. F. et al. (2006). Prevalence of monoclonal gammopathy of undetermined significance. New England Journal of Medicine*,* 354 *(*13*),* 1362–1369. 10.1056/NEJMoa054494; 16571879

[4] Kyle, R. A., Larson, D. R., Thernau, T. M., Dispenzieri, A., Kumar, S. et al. (2018). Long-term follow-up of monoclonal gammopathy of undetermined significance. New England Journal of Medicine*,* 378 *(*3*),* 241–249. 10.1056/NEJMoa1709974; 29342381 PMC5852672

[5] Rajkumar, S. V., Dimopoulos, M. A., Palumbo, A., Blade, J., Merlini, G. et al. (2014). International myeloma working group updated criteria for the diagnosis of multiple myeloma. Lancet Oncology*,* 15 *(*12*),* e538–e548. 10.1016/S1470-2045(14)70442-5; 25439696

[6] Gozzetti, A., Guarnieri, A., Zamagni, E., Zakharova, E., Coriu, D. et al. (2022). Monoclonal gammopathy of renal significance (MGRS): Real-world data on outcomes and prognostic factors. American Journal of Hematology*,* 97 *(*7*),* 877–884. 10.1002/ajh.26566; 35389534 PMC9324084

[7] Ocio, E. M., Richardson, P. G., Rajkumar, S. V., Palumbo, A., Mateos, M. V. et al. (2014). New drugs and novel mechanisms of action in multiple myeloma in 2013: A report from the international myeloma working group (IMWG). Leukemia*,* 28 *(*3*),* 525–542. 10.1038/leu.2013.350; 24253022 PMC4143389

[8] Kumar, S. K., Rajkumar, S. V., Dispenzieri, A., Lacy, M. Q., Hayman, S. R. et al. (2008). Improved survival in multiple myeloma and the impact of novel therapies. Blood*,* 111 *(*5*),* 2516–2520. 10.1182/blood-2007-10-116129; 17975015 PMC2254544

[9] Palumbo, A., Falco, P., Falcone, A., Benevolo, G., Canepa, L. et al. (2009). Melphalan, prednisone, and lenalidomide for newly diagnosed myeloma: Kinetics of neutropenia and thrombocytopenia and time-to-event results. Clinical Lymphoma Myeloma*,* 9 *(*2*),* 145–150. 10.3816/CLM.2009.n.035; 19406725

[10] Castillo, J. J., Jurczyszyn, A., Brozova, L., Crusoe, E., Czepiel, J. et al. (2017). IgM myeloma: A multicenter retrospective study of 134 patients. American Journal of Hematology*,* 92 *(*8*),* 746–751. 10.1002/ajh.24753; 28383205

[11] Jurczyszyn, A., Radocha, J., Davila, J., Fiala, M. A., Gozzetti, A. et al. (2018). Prognostic indicators in primary plasma cell leukaemia: A multicentre retrospective study of 117 patients. British Journal Haematology*,* 180 *(*6*),* 831–839. 10.1111/bjh.15092; 29315478

[12] Gozzetti, A., Cerase, A. (2014). Novel agents in CNS myeloma treatment. Central Nervous System Agents Medicinal Chemistry*,* 14 *(*1*),* 23–27. 10.2174/1871524914999140818111514; 25134940

[13] Gozzetti, A., Cerase, A., Lotti, F., Rossi, D., Palumbo, A. et al. (2012). Extramedullary intracranial localization of multiple myeloma and treatment with novel agents: A retrospective survey of 50 patients. Cancer*,* 118 *(*6*),* 1574–1584. 10.1002/cncr.26447; 21932386

[14] Flores-Montero, J., Sanoja-Flores, L., Paiva, B., Puig, N., Garcia-Sanchez, O. et al. (2017). Next generation flow for highly sensitive and standardized detection of minimal residual disease in multiple myeloma. Leukemia*,* 31 *(*10*),* 2094–2103. 10.1038/leu.2017.29; 28104919 PMC5629369

[15] Kumar, S., Paiva, B., Anderson, K. C., Durie, B., Landgren, O. et al. (2016). International myeloma working group consensus criteria for response and minimal residual disease assessment in multiple myeloma. Lancet Oncology*,* 17 *(*8*),* e328–e346. 10.1016/S1470-2045(16)30206-6; 27511158

[16] Munshi, N. C., Avet-Loiseau, H., Anderson, K. C., Neri, P., Paiva, B. et al. (2020). A large meta-analysis establishes the role of MRD negativity in long-term survival outcomes in patients with multiple myeloma. Blood Advances*,* 4 *(*23*),* 5988–5999. 10.1182/bloodadvances.2020002827; 33284948 PMC7724898

[17] Gozzetti, A., Raspadori, D., Bacchiarri, F., Sicuranza, A., Pacelli, P. et al. (2020). Minimal residual disease in multiple myeloma: State of the art and applications in clinical practice. Journal Personalized Medicine*,* 10 *(*3*),* 120. 10.3390/jpm10030120; 32927719 PMC7565263

[18] Zinzani, P. L., Zompatori, M., Bendandi, M., Battista, G., Fanti, S. et al. (1996). Monitoring bulky mediastinal disease with gallium-67, CT-scan and magnetic resonance imaging in Hodgkin’s disease and high-grade non-Hodgkin’s lymphoma. Leukemia & Lymphoma*,* 22 *(*1–2*),* 131–135. 10.3109/10428199609051740; 8724540

[19] Lecouvet, F. E., Vekemans, M. C., van den Berghe, T., Verstraete, K., Kirchgesner, T. et al. (2022). Imaging of treatment response and minimal residual disease in multiple myeloma: State of the art WB-MRI and PET/CT. Skeletal Radiology*,* 51 *(*1*),* 59–80. 10.1007/s00256-021-03841-5; 34363522 PMC8626399

[20] Hillengass, J., Usmani, S., Rajkumar, S. V., Durie, B. G. M., Mateos, M. V. et al. (2019). International myeloma working group consensus recommendations on imaging in monoclonal plasma cell disorders. Lancet Oncology*,* 20 *(*6*),* e346. 10.1016/S1470-2045(19)30309-2; 31162104

[21] Thoren, K. L. (2018). Mass spectrometry methods for detecting monoclonal immunoglobulins in multiple myeloma minimal residual disease. Seminars Hematology*,* 55 *(*1*),* 41–43. 10.1053/j.seminhematol.2018.02.008; 29759153

[22] Chapman, J. R., Thoren, K. L. (2020). Tracking of low disease burden in multiple myeloma: Using mass spectrometry assays in peripheral blood. Best Practice Research Clinical Haematology*,* 33 *(*1*),* 101142. 10.1016/j.beha.2020.101142; 32139008

[23] Mills, J. R., Kohlhagen, M. C., Dasari, S., Vanderboom, P. M., Kyle, R. A. et al. (2016). Comprehensive assessment of M-proteins using nanobody enrichment coupled to MALDI-TOF mass spectrometry. Clinical Chemistry*,* 62 *(*10*),* 1334–1344. 10.1373/clinchem.2015.253740; 27540026

[24] Barnidge, D. R., Tschumper, R. C., Theis, J. D., Snyder, M. R., Jelinek, D. F. et al. (2014). Monitoring M-proteins in patients with multiple myeloma using heavy-chain variable region clonotypic peptides and LC-MS/MS. Journal Proteome Research*,* 13 *(*4*),* 1905–1910. 10.1021/pr5000544; 24552626

[25] Bergen, H. R. III, Dasari, S., Dispenzieri, A., Mills, J. R., Ramirez-Alvarado, M. et al. (2016). Clonotypic light chain peptides identified for monitoring minimal residual disease in multiple myeloma without bone marrow aspiration. Clinical Chemistry*,* 62 *(*1*),* 243–251. 10.1373/clinchem.2015.242651; 26430073 PMC5003037

[26] Barnidge, D. R., Dasari, S., Ramirez-Alvarado, M., Fontan, A., Willrich, M. A. et al. (2014). Using mass spectrometry to monitor monoclonal immunoglobulins in patients with a monoclonal gammopathy. Journal Proteome Research*,* 13 *(*3*),* 1419–1427. 10.1021/pr400985k; 24467232

[27] Zajec, M., Jacobs, J. F. M., Groenen, P. J. T. A., de Kat Angelino, C. M., Stingl, C. et al. (2018). Development of a targeted mass-spectrometry serum assay to quantify M-protein in the presence of therapeutic monoclonal antibodies. Journal Proteome Research*,* 17 *(*3*),* 1326–1333. 10.1021/acs.jproteome.7b00890; 29424538

[28] Martins, C. O., Huet, S., Yi, S. S., Ritorto, M. S., Landgren, O. et al. (2020). Mass spectrometry-based method targeting Ig variable regions for assessment of minimal residual disease in multiple myeloma. Journal Molecular Diagnostics*,* 22 *(*7*),* 901–911. 10.1016/j.jmoldx.2020.04.002; 32302778 PMC7338887

[29] Eveillard, M., Rustad, E., Roshal, M., Zhang, Y., Ciardiello, A. et al. (2020). Comparison of MALDI-TOF mass spectrometry analysis of peripheral blood and bone marrow-based flow cytometry for tracking measurable residual disease in patients with multiple myeloma. British Journal Haematology*,* 189 *(*5*),* 904–907. 10.1111/bjh.16443; 32026474 PMC7275888

[30] Mills, J. R., Barnidge, DR., Dispenzieri, A., Murray, DL. (2017). High sensitivity blood-based M-protein detection in sCR patients with multiple myeloma. Blood Cancer Journal*,* 7 *(*8*),* e590. 10.1038/bcj.2017.75; 28841203 PMC5596386

[31] Derman, B. A., Stefka, A. T., Jiang, K., McIver, A., Kubicki, T. et al. (2021). Measurable residual disease assessed by mass spectrometry in peripheral blood in multiple myeloma in a phase II trial of carfilzomib, lenalidomide, dexamethasone and autologous stem cell transplantation. Blood Cancer Journal*,* 11 *(*2*),* 19. 10.1038/s41408-021-00418-2; 33563912 PMC7873068

[32] Heitzer, E., Haque, I. S., Roberts, C. E. S., Speicher, M. R. (2019). Current and future perspectives of liquid biopsies in genomics driven oncology. Nature Reviews Genetics*,* 20 *(*2*),* 71–88. 10.1038/s41576-018-0071-5; 30410101

[33] Pantel, K., Alix-Panabières, C. (2019). Liquid biopsy and minimal residual disease—latest advances and implications for cure. Nature Reviews Clinical Oncolology*,* 16 *(*7*),* 409–424. 10.1038/s41571-019-0187-3; 30796368

[34] Kis, O., Kaedbey, R., Chow, S., Danesh, A., Dowar, M. et al. (2017). Circulating tumour DNA sequence analysis as an alternative to multiple myeloma bone marrow aspirates. Nature Communication*,* 8*,* 15086. 10.1038/ncomms15086; 28492226 PMC5437268

[35] Manier, S., Park, J., Capelletti, M., Bustoros, M., Freeman, S. S. et al. (2018). Whole-exome sequencing of cell-free DNA and circulating tumor cells in multiple myeloma. Nature Communication*,* 9 *(*1*),* 1691. 10.1038/s41467-018-04001-5 PMC5923255 29703982

[36] Vij, R., Mazumder, A., Klinger, M., O’Dea, D., Paasch, J. et al. (2014). Deep sequencing reveals myeloma cells in peripheral blood in majority of multiple myeloma patients. Clinical Lymphoma Myeloma Leukemia*,* 14 *(*2*),* 131–139. 10.1016/j.clml.2013.09.013; 24629890

[37] Sanoja-Flores, L., Flores-Montero, J., Garcés, J. J., Paiva, B., Puig, N. et al. (2018). Next generation flow for minimally-invasive blood characterisation of MGUS and multiple myeloma at diagnosis based on circulating tumor plasma cells (CTPC). Blood Cancer Journal*,* 8 *(*12*),* 117. 10.1038/s41408-018-0153-9; 30455467 PMC6242818

[38] Sanoja-Flores, L., Flores-Montero, J., Puig, N., Contreras-Sanfeliciano, T., Pontes, R. et al. (2019). Blood monitoring of circulating tumor plasma cells by next generation flow in multiple myeloma after therapy. Blood*,* 134 *(*24*),* 2218–2222. 10.1182/blood.2019002610; 31697808 PMC6966491

[39] Wang, N., Tesfaluul, N., Li, J., Gao, X., Liu, S. et al. (2019). Enrichment of circulating myeloma cells by immunomagnetic beads combined with flow cytometry for monitoring minimal residual disease and relapse in patients with multiple myeloma. Annals Hematology*,* 98 *(*12*),* 2769–2780. 10.1007/s00277-019-03833-5; 31748925

